# O-antigen biosynthesis gene clusters of *Escherichia albertii*: their diversity and similarity to *Escherichia coli* gene clusters and the development of an O-genotyping method

**DOI:** 10.1099/mgen.0.000314

**Published:** 2019-11-18

**Authors:** Tadasuke Ooka, Kazuko Seto, Yoshitoshi Ogura, Keiji Nakamura, Atsushi Iguchi, Yasuhiro Gotoh, Mikiko Honda, Yoshiki Etoh, Tetsuya Ikeda, Wakana Sugitani, Takayuki Konno, Kimiko Kawano, Naoko Imuta, Kiyotaka Yoshiie, Yukiko Hara-Kudo, Koichi Murakami, Tetsuya Hayashi, Junichiro Nishi

**Affiliations:** ^1^​ Department of Microbiology, Graduate School of Medical and Dental Sciences, Kagoshima University, 8-35-1 Sakuragaoka, Kagoshima 890-8544, Japan; ^2^​ Osaka Institute of Public Health, 1-3-69 Nakamichi, Higasinari-ku, Osaka 537-0025, Japan; ^3^​ Department of Bacteriology, Faculty of Medical Sciences, Kyushu University, 3-1-1 Maidashi, Higashi-ku, Fukuoka 812-8582, Japan; ^4^​ Department of Animal and Grassland Sciences, Faculty of Agriculture, University of Miyazaki, 1-1 Gakuen-kibanadai-nishi, Miyazaki 889-2192, Japan; ^5^​ Fukuoka City Institute of Hygiene and the Environment, 2-1-34 Jigyohama, Chuo-ku, Fukuoka 810-0065, Japan; ^6^​ Fukuoka Institute of Health and Environmental Sciences, 39 Mukaizano, Dazaifu, Fukuoka 818-0135, Japan; ^7^​ Hokkaido Institute of Public Health, Kita-19, Nishi-12, Kita-ku, Sapporo 060-0819, Japan; ^8^​ Kumamoto City Environmental Research Institute, 404-1, Ezumachi Tokorojima, Higashi-ku, Kumamoto 862-0946, Japan; ^9^​ Akita Prefectural Research Center for Public Health and Environment, 6-6 Senshu Kubota-machi, Akita 010-0874, Japan; ^10^​ Miyazaki Prefectural Institute for Public Health and Environment, 2-3-2 Gakuen-kibanadai-nishi, Miyazaki 889-2155, Japan; ^11^​ National Institute of Health Sciences, 3-25-26, Tonomachi, Kawasaki-ku, Kawasaki 210-9501, Japan; ^12^​ Infectious Disease Surveillance Center, National Institute of Infectious Diseases, 4-7-1 Gakuen, Musashi-murayama, Tokyo 208-0011, Japan

**Keywords:** *Escherichia albertii*, O-antigen gene cluster, genotyping, genome, phylogeny

## Abstract

*
Escherichia albertii* is a recently recognized human enteropathogen that is closely related to *Escherichia coli *. In many Gram-negative bacteria, including *E. coli *, O-antigen variation has long been used for the serotyping of strains. In *E. albertii *, while eight O-serotypes unique to this species have been identified, some strains have been shown to exhibit genetic or serological similarity to known *E. coli */* Shigella* O-serotypes. However, the diversity of O-serotypes and O-antigen biosynthesis gene clusters (O-AGCs) of *E. albertii* remains to be systematically investigated. Here, we analysed the O-AGCs of 65 *E. albertii* strains and identified 40 *E. albertii* O-genotypes (EAOgs) (named EAOg1–EAOg40). Analyses of the 40 EAOgs revealed that as many as 20 EAOgs exhibited significant genetic and serological similarity to the O-AGCs of known *E. coli */* Shigella* O-serotypes, and provided evidence for the inter-species horizontal gene transfer of O-AGCs between *E. albertii* and *E. coli *. Based on the sequence variation in the *wzx* gene among the 40 EAOgs, we developed a multiplex PCR-based O-genotyping system for *E. albertii* (EAO-genotyping PCR) and verified its usefulness by genotyping 278 *E. albertii* strains from various sources. Although 225 (80.9 %) of the 278 strains could be genotyped, 51 were not assigned to any of the 40 EAOgs, indicating that further analyses are required to better understand the diversity of O-AGCs in *E. albertii* and improve the EAO-genotyping PCR method. A phylogenetic view of *E. albertii* strains sequenced so far is also presented with the distribution of the 40 EAOgs, which provided multiple examples for the intra-species horizontal transfer of O-AGCs in *E. albertii *.

## Data Summary

The assembled and read sequences of 11 *Escherichia albertii* strains obtained in this study have been deposited in GenBank/EMBL/DDBJ under the BioProject accession number PRJDB8401. The annotated sequences of O-antigen biosynthesis gene clusters have been deposited in GenBank/EMBL/DDBJ under accession numbers LC494303–LC494359.

**Impact Statement**

*
Escherichia albertii* is a close relative of *Escherichia coli* and has been frequently misidentified as enteropathogenic *E. coli* (EPEC) due to their phenotypic and genetic similarity and their possession of a similar type III secretion system (T3SS). The clinical importance of *E. albertii* is increasingly being recognized, as this species causes outbreaks of gastroenteritis, and some strains produce Shiga toxins, similar to enterohaemorrhagic *E. coli* (EHEC). Rapid strain typing systems useful for outbreak investigation and surveillance, such as O-serotyping, which is widely used for *E. coli *, need to be developed. However, the diversity of O-serotypes and O-antigen biosynthesis gene clusters (O-AGCs) of *E. albertii* is largely unknown. This study identified 40 *E. albertii* O-genotypes by analysing the O-AGCs of 65 *E. albertii* strains and provides what is believed to be the first insight into the diversity of *E. albertii* O-AGCs, as well as evidence for the horizontal transfer of O-AGCs within *E. albertii *, and between *E. albertii* and *E. coli *. Moreover, based on these results, a multiplex PCR-based O-genotyping system for *E. albertii* was developed, which could contribute to the improvement and promotion of epidemiological studies of this understudied pathogen.

## Introduction

*Escherichia albertii* is a recently recognized human enteropathogen and an avian pathogen responsible for epidemic mortality [1–3]. Several outbreaks caused by this micro-organism have recently been reported [4–6]. *E. albertii* strains carrying Shiga toxin (Stx) genes (*stx2a* or *stx2f*) have been identified [3, 7, 8], indicating that there is a significant risk of severe infections caused by this species. Another important aspect is that *E. albertii* infections have been underestimated because they have been frequently misidentified as enteropathogenic or enterohaemorrhagic *Escherichia coli* (EPEC or EHEC, respectively) due to their similarity in phenotypic and genetic features, such as biochemical properties and the possession of the locus of enterocyte effacement (LEE), encoding a type III secretion system (T3SS) [3]. Although genome sequencing analyses of more than 50 *E. albertii* strains have been reported [9–13], the genomic diversity of this species is yet to be fully understood.

O-antigen is a lipopolysaccharide component of the outer membrane of Gram-negative bacteria. The chemical compositions and structures are highly variable even in the same species [14, 15], which have long been used for the serotyping of strains. In *E. coli *, O-antigen biosynthesis gene clusters (O-AGCs) are located between the colonic acid biosynthesis gene cluster (the *wca* genes) and the *his* operon on the chromosome, with a few exceptions [16, 17]. O-antigen biosynthesis genes fall into three classes: (i) genes for nucleotide sugar biosynthesis, (ii) genes encoding sugar transferase, and (iii) genes for O-unit translocation and chain synthesis [18]. The variation in the repertoire of these genes in O-AGCs is responsible for the differences in O-antigen structure and, therefore, in O-serotype. Structural and serological changes in O-antigens can occur via point mutations in the genes in O-AGCs, such as glycosyltransferase genes, or by acquisition of O-antigen modification genes [19, 20].

The presence of genetic similarity between some *E. albertii* and *E. coli* O-AGCs has been suggested by an analysis of O-AGCs of published *E. albertii* genomes [21], while eight O-serotypes (named EAO1–EAO8) unique to *E. albertii* have been identified along with their chemical structures [21–26]. It has also been reported that an environmental *E. albertii* isolate (strain DM104) serologically cross-reacts with the antiserum of *Shigella dysenteriae* type 4 [27], and that some *E. albertii* strains express the O-antigen of *Shigella boydii* serotype 13 [28]. However, to better understand the diversity of *E. albertii* O-serotypes and the genetic variation underlying their diversity and similarity to *E. coli* O-serotypes, a systematic, large-scale analysis of *E. albertii* O-AGCs is required.

In this study, we performed detailed analyses of the O-AGCs of 65 *E. albertii* strains, including EAO1–EAO8, and those of 57 genome-sequenced strains, to investigate the diversity of *E. albertii* O-AGCs. Inter-species comparison of O-AGCs with known *E. coli* O-AGCs and those of *E. coli* relatives was also performed to understand the serological and genetic similarity between *E. albertii* and *E. coli* O-serotypes. Based on the results, we attempted to develop a multiplex PCR-based O-genotyping system for *E. albertii* (EAO-genotyping PCR) and to provide a current view on the diversity of O-serotypes in the phylogenetic context of *E. albertii* strains.

## Methods

### Bacterial strains

The 65 *E. albertii* strains analysed in this study are listed in Table S1, (available with the online version of this article) along with detailed information on each strain. Among these strains, 57 were genome-sequenced strains, including 30 that were sequenced in our previous study [9, 10], 16 for which the genome sequences were obtained from the GenBank genome database [11–13] and 11 that were sequenced in this study (Table S1). The strain information for the 92 *E. albertii* strains used for the evaluation of EAO-genotyping PCR is provided in Table S2. The *E. albertii* strains analysed were cultured overnight in lysogeny broth (LB; Nippon Becton Dickinson) at 37 °C with shaking.

### Genome sequencing of 11 *E. albertii* strains

Genomic DNA was purified from a 2 ml overnight culture using the DNeasy blood and tissue kit (Qiagen), according to the manufacturer’s instructions. Sequencing libraries for each strain were prepared using the Nextera XT DNA sample prep kit (Illumina) to obtain paired-end sequences (300 bp ×2) on an Illumina MiSeq instrument. Draft genome sequences were obtained by assembling the read sequences using Platanus v1.1.4 with default parameters [29]. The sequencing status of each strain is shown in Table S3.

### Identification and annotation of O-AGCs of *E. albertii* strains

O-AGCs were identified by blastn search using the *galF* and *gnd* sequences as queries, with an *E* value threshold of 0.01. Protein encoding sequences and their functions were predicted using the Microbial Genome Annotation Pipeline (MiGAP; http://www.migap.org) which has been recently closed, followed by manual curation using the *in silico* Molecular Cloning Genomics Edition software v7.29L (IMC-GE; In Silico Biology). Domain searches were performed using the Pfam program v32, with an *E* value threshold of 0.01 [30].

### Intra- and inter-species comparison of O-AGCs

For this analysis, we obtained the O-AGC sequences of 8 known *E. albertii* serotypes [21–23, 26], 201 known *E. coli* O-serotypes [16, 17, 31] and 19 known *Shigella* serotypes [32] from the NCBI database (Table S4). In addition, we analysed the publicly available genome sequences of three *Escherichia fergusonii* strains and eight strains belonging to cryptic *Escherichia* clades, and identified and annotated their O-AGCs as described above. The accession numbers of the O-AGC sequences and genome sequences used for comparative analysis are listed in Table S4 (* Escherichia */* Shigella* species). Sequence comparison of the O-AGCs was performed by blastn using the nucleotide sequences of each protein.

### Agglutination test using the antisera for known *E. coli* O-antigens

O-serotype cross-reactivity was determined by the slide agglutination test and/or the microtitre plate method using the *E. coli* antisera set 1 (Denka Seiken), the pooled and single antisera against all known *E. coli* O-serotypes (SSI Diagnostica), and the *S. boydii* type 13 antiserum (Denka Seiken), according to the manufacturers’ instructions. The SSI Diagnostica set included antisera against *E. coli* O1 to O187, excluding five serotypes (O31, O47, O67, O94 and O122; these serotypes are missing in the current serotyping scheme). When a strain was weakly agglutinated compared with the reference strain or agglutinated with multiple antisera, we performed titration tests using microtitre plates. Weak agglutination was defined if the titre was four times lower than that of the reference strain.

### Development of a multiplex EAO-genotyping PCR

Based on the sequence variation in the *wzx* genes of all *E. albertii* and other *Escherichia */* Shigella* species, we designed three primer sets, containing 12, 14 and 14 primer pairs (from the first to third sets), to specifically detect each of the 40 *E. albertii* O-genotypes (EAOgs) (Table S5). Previously designed primer pairs targeting an *E. albertii *-specific region [9] were also included in each primer set as a positive control and a genetic marker of *E. albertii* (E_al_1_OF in the first set; E_al_1_NF in the second and third sets). Template DNA for PCR was prepared by the alkaline-boiling method, as described previously [33]. KOD -Multi and Epi- DNA polymerase (Toyobo) was used for PCR. Each reaction mixture (15 μl) contained 1 μl template DNA, 4.5 μM each primer and 0.3 U polymerase. PCR was conducted with initial denaturation for 2 min at 94 °C, followed by 25 cycles of 10 s at 94 °C, 30 s at 60 °C and 30 s at 68 °C. PCR products were analysed by agarose gel electrophoresis using 2 % agarose S (Nippon Gene).

### *In silico* EAO-genotyping

*In silico* EAO-genotyping was performed for the strains registered in the EnteroBase website v1.1.2. (https://enterobase.warwick.ac.uk [34]). From the 274 strains registered as *E. albertii* (accessed on 9 September 2019), 87 strains were excluded due to the overlap with the strains used for detailed analysis in this study or the lack of genome sequence information. In addition, one strain was excluded due to a low completeness (<95 %) and a high contamination (>5 %) as estimated by CheckM [35], and a high level of fragmentation (longest contig<100 kb). The final set of *E. albertii* strains used for *in silico* EAO-genotyping (*n*=186) is listed in Table S6. *In silico* EAO-genotyping was performed by blastn search using the nucleotide sequences of the 42 primer pairs (including 2 primer pairs targeting a genetic marker of *E. albertii *) designed for EAO-genotyping PCR as queries (Table S5). Only a perfect match was considered.

### Phylogenetic analyses

Sequence comparisons and phylogenetic analyses of the *wzx*/*wzy* and *wzm*/*wzt* genes of 40 *E. albertii* EAOs and those of known *E. coli */* Shigella* O-serotypes and other *Escherichia* species (Table S4) were performed using mega6 software [36]. In brief, the nucleic acid sequences of each gene were aligned by ClustalW with default parameters. A phylogenetic tree was reconstructed with the neighbour-joining method using the p-distance model to calculate nucleotide distance. Bootstrap analysis with 1000 replicates was performed to evaluate the significance of internal branches. For the reconstruction of a core gene-based phylogenetic tree, the genome assemblies of 57 strains used for detailed analysis of O-AGCs and 186 strains used for *in silico* EAO-genotyping were annotated using Prokka [37], and core genes (*n*=2128) were identified using Roary v3.11.2 [38] with a 90 % amino acid sequence identity cut-off. Core gene SNPs (*n*=94 287) were extracted using the core gene alignment tool in Roary and used as inputs for maximum-likelihood (ML) inference with RAxML v8 [39]. The ML tree was displayed and annotated using iTOL v4 (https://itol.embl.de) [40]. The tree was rooted by the mid-point rooting method and the confidence of each branch was estimated by bootstrap with 200 replications. Identical *E. albertii* genomes showing no SNPs were deduplicated (excluded strains are indicated in Table S6).

## Results

### Sequence analysis of the O-AGCs from 65 *E. albertii* strains

To enrich the genome sequence and strain resources of *E. albertii* that can be used for the detailed analysis of O-AGCs and serotypes, we newly sequenced 11 *E. albertii* strains in this study. By adding these sequences to the 46 previously sequenced genomes and the 8 O-AGC sequences previously reported as EAO1–EAO8, we analysed the O-AGCs of 65 *E. albertii* strains from various sources, including humans, birds, cats and the environment (Table S1).

### General features and genotypes of the *E. albertii* O-AGCs

**Fig. 1.**
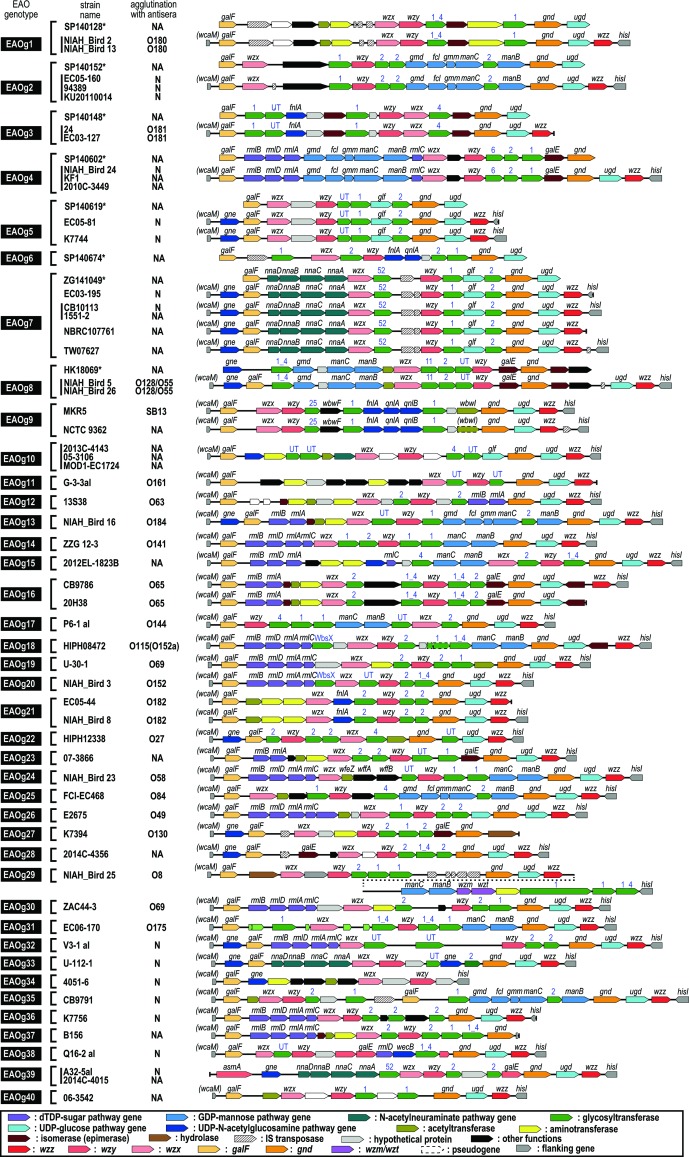
Genetic structures of the 40 *E. albertii* O-genotypes identified. Genotypes, strain names and results of the agglutination test using antisera against known *E. coli */* Shigella* O-serotypes (N, no agglutination; NA, not applicable) are indicated on the left side. The structures of O-AGCs were deduced from the genome sequences of each strain except for the eight strains indicated by asterisks. Only the sequences of O-AGCs are available for the eight strains. For putative glycosyltransferases (indicated by green), types of domain families are indicated instead of gene names (UT, untypable).

In 47 of the 65 strains analysed, O-AGCs were located between the truncated *wcaM* gene and the *hisI* gene. In 10 strains, either the *wcaM* or the *hisI* gene was identified, but the opposite boundary of O-AGC was not identified due to the interruption of corresponding contig sequences in their draft genomes (Fig. 1). The remaining eight strains were the previously reported strains of EAO1–EAO8, for which only the sequences between the *galF* and *ugd* genes were available. The sizes of *E. albertii* O-AGCs ranged from 13 to 30 kb. The mean G+C content was 37.8 mol% (ranging from 35.1 to 42.5 mol%). Based on gene organization, the 65 O-AGCs were grouped into 40 clearly distinguishable genotypes, named EAOg1–EAOg40 (Fig. 1, Table S1). EAO1–EAO8 are referred to as EAOg1–EAOg8, respectively, in this manuscript. The O-AGC corresponding to EAO6 (now referred to as EAOg6) was not found in the 57 genome-sequenced strains.

### Nucleotide sugar biosynthesis genes

The *rmlBDAC* genes, required for the synthesis of deoxythymidine diphosphate (dTDP)-l-rhamnose (the precursor of l-rhamnose) by the dTDP-sugar biosynthesis pathway, were present in 12 O-genotypes (Fig. 1). Three of the genotypes (EAOg12, EAOg13 and EAOg16) contained only the *rmlBA* genes. Similar to the O-AGCs of *E. coli* O63, O184 and O65, which are homologous to each of the three EAOgs described below, it is likely that the dTDP-6-deoxy-d-xylo-4-hexulose 3,5-epimerase (encoded by *rmlC*) and dTDP-4-dehydrorhamnose reductase (*rmlD*) genes are located outside the O-AGCs. The *nnaDBCA* genes for the synthesis of cytidine monophosphate-*N*-acetylneuraminate (CMP-NeuNAc) were found in three O-genotypes (EAOg7, EAOg33 and EAOg39). The *fnlA* and *qnlBC* genes for the synthesis of UDP-*N*-acetyl-l-quinovosamine (UDP-l-QuiNAc) were found in EAOg9. The remaining 24 O-genotypes contained no nucleotide sugar biosynthesis genes or only a limited number of genes for the biosynthesis of certain nucleotide sugars, suggesting that, in these O-genotypes, nucleotide sugars or their precursors to be incorporated into O-antigen are supplied via the functions of genes located outside the O-AGCs.

### Glycosyltransferase genes

All the genotypes except EAOg34 contained two to six genes encoding putative glycosyltransferases (132 glycosyltransferase genes in total). A Pfam search of the 132 gene products identified 9 types of glycosyltransferase-related domains (Table S7; 18 were untypable), with families 2 (PF00535), 1 (PF00534) and 1_4 (PF13692) being the most widely distributed (51, 37 and 13 genes, respectively).

### Genes for O-antigen processing

For O-unit processing and conversion of the O-unit to O-antigen, all the O-genotypes contained the *wzx*/*wzy* gene set. EAOg29 additionally contained the *wzm*/*wzt* gene set.

### Comparison of *E. albertii* O-AGCs with known *Escherichia */* Shigella* O-AGCs

**Fig. 2.**
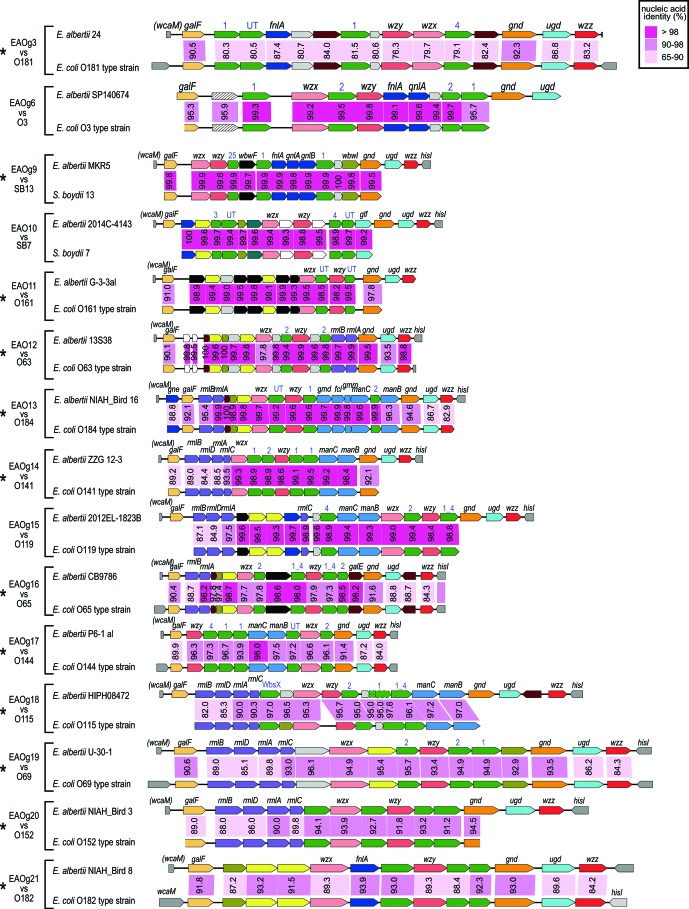

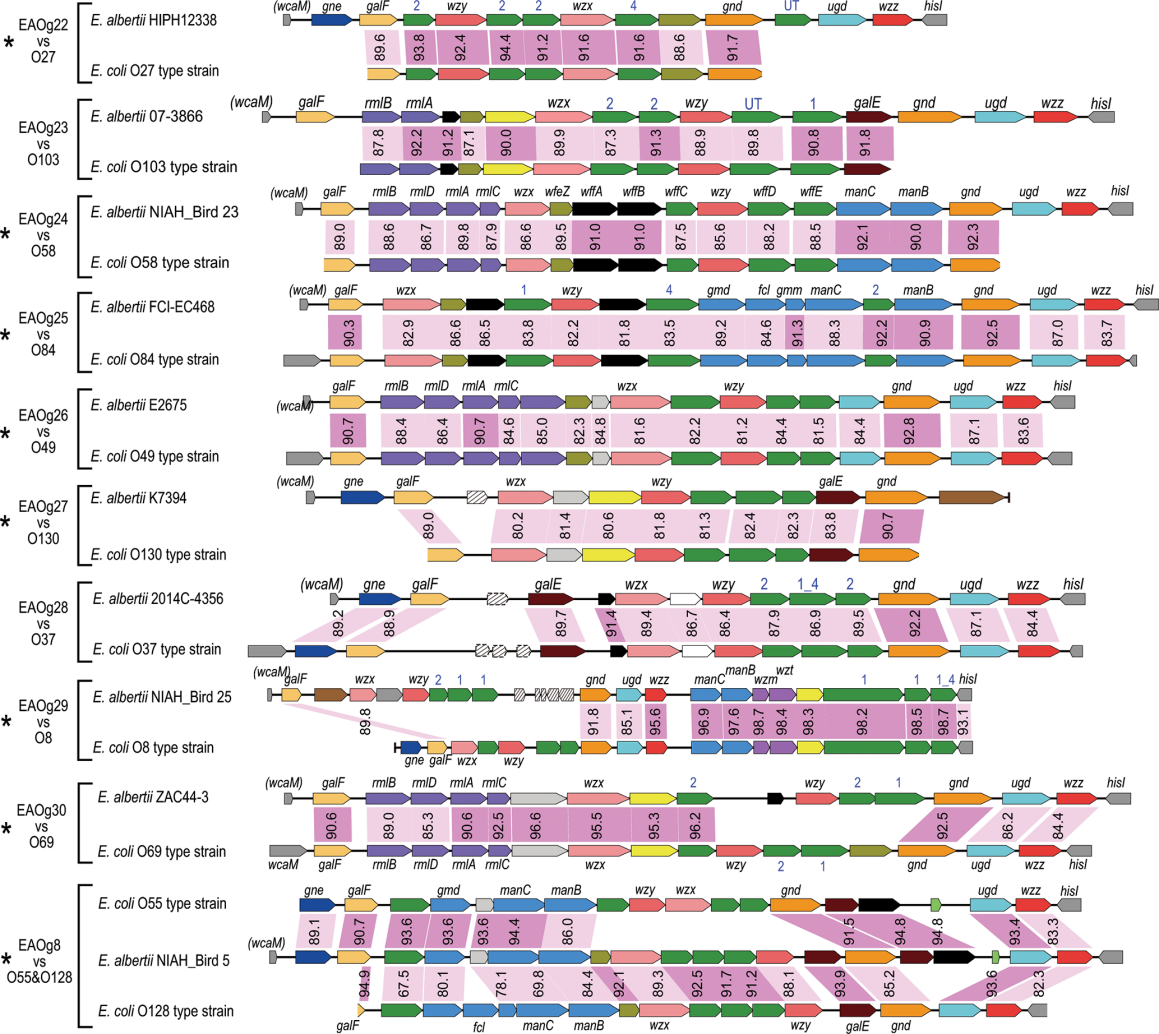
Comparison of the O-AGCs of EAOgs with homologous O-AGCs of known *E. coli/Shigella* O-serotypes. Shading and numbers between O-AGCs indicate the nucleotide sequence identities (%) of each gene. EAOgs indicated by asterisks (*n*=20) were found to be serologically cross-reactive with each *E. coli/Shigella* counterpart. Although EAOg30 cross-reacts with *E. coli* O69 and weakly with O124, its O-AGC shows similarity only to that of *E. coli* O69. EAOg8 exhibits significant similarity to *E. coli* O55 and O128 and cross-reacts with both *E. coli* O-serotypes. In the five EAOgs without asterisks, the cross-reactivity to their counterparts could not be examined due to a lack of *E. albertii* strains in our laboratory.

We analysed the structural similarity of the O-AGCs of the 40 EAOgs to those of known *Escherichia */* Shigella* O-serotypes. The O-AGCs of 20 EAOgs (EAOg3, EAOg6 and EAOg9–26) were significantly similar to those of some *Escherichia */* Shigella* serotypes in terms of gene organization and nucleotide sequence identity [most genes showed >80 % nucleotide sequence identity (83–99 % on average) to the gene of each counterpart] (Table S1, Fig. 2). Among the 20 EAOgs, eight (EAOg6 and EAOg9–EAOg15) exhibited particularly high sequence similarity [most genes showed >98 % nucleotide sequence identity (>96 % on average)] to the counterparts of *E. coli* or *S. boydii* (Table S1, Fig. 2).

In contrast to the 20 EAOgs, most genes in 3 EAOgs (EAOg27–EAOg29) contained the same set of genes as counterparts in *E. coli *, but the nucleotide sequence identities were 83–89 % on average (Table S1, Fig. 2). Two EAOgs (EAOg8 and EAOg30) showed sequence similarity to only parts of the O-AGCs of *E. coli* O128/O55 and O69, respectively (Fig. 2). The remaining 15 EAOgs (EAOg1, EAOg2, EAOg4, EAOg5, EAOg7 and EAOg31–EAOg40) showed no significant similarity to the O-AGCs of known *E. coli */* Shigella* serotypes, except for local similarities (e.g. between the genes encoding proteins of the same family). No significant similarity was observed between the O-AGCs of the 40 EAOgs and those of genome-sequenced strains belonging to *E. fergusonii* and the cryptic clades of *Escherichia* species.

### Serological cross-reactivity of *E. albertii* O-antigens to known *E. coli* O-serotypes and *S. boydii* type 13

As many of the 40 EAOgs showed high similarity to the O-AGCs of known *E. coli */* Shigella* serotypes, we analysed the serological cross-reactivity of the O-antigens of *E. albertii* to *E. coli */* Shigella* O-antigens using antisera against 182 known *E. coli* O-serotypes and those of *Shigella* species. In this analysis, only 42 *E. albertii* strains (belonging to 33 EAOgs) were used, because the remaining 14 strains (belonging to EAOg6, EAOg10, EAOg15, EAOg23, EAOg28, EAOg37 and EAOg40) were not available in our laboratories. As shown in Table S1 and Fig. 1, 1 and 22 of the 33 O-genotypes tested were agglutinated with antisera against known *E. coli* O-serotypes or *S. boydii* type 13, suggesting that the O-antigens of these O-genotypes serologically cross-reacted with *E. coli* or *Shigella* O-antigens. EAOg18 was agglutinated with the *E. coli* O115 antisera from both SSI Diagnostica and Denka Seiken, and with the *E. coli* O152a antiserum from SSI Diagnostica (not with the antiserum from Denka Seiken). EAOg8 was also agglutinated with both *E. coli* O55 and O128 antisera. For EAOg13, which was agglutinated with the *E. coli* O184 antiserum, weak agglutination was also observed with the *E. coli* O103 antiserum. Similarly, EAOg30, which was agglutinated with the *E. coli* O69 antiserum, exhibited weak agglutination with the *E. coli* O124 antiserum. The remaining 11 EAOgs were not agglutinated with any *E. coli* O-serotype antiserum. *E. albertii* strains belonging to the same O-genotype exhibited the same agglutination pattern.

### Correlation of the genetic similarity of O-genotypes and their serological cross-reactivity to known O-serotypes of *Escherichia/Shigella* species

We examined the correlation between the structural similarity of O-AGCs and the serological cross-reactivity to known *Escherichia */* Shigella* O-serotypes. Among 25 EAOgs (EAOg3, EAOg6 and EAOg8–30) that showed notable similarities to known *E. coli */* Shigella* serotypes, 5 (EAOg6, EAOg10, EAOg15, EAOg23 and EAOg28) were unable to undergo serological examination. However, all of the remaining 20 EAOgs tested showed serological cross-reactivity to the *E. coli */* Shigella* counterparts (the serotypes that showed structural similarities). Thus, the structural similarity of O-AGCs between *E. albertii* and *Escherichia */* Shigella* is well correlated with the serological cross-reactivity between these O-serotypes, suggesting that the five EAOgs that could not be tested probably cross-reacted with their *E. coli* counterparts. The exceptions observed were EAOg1 and EAOg31, which showed cross-reactivity with *E. coli* O180 and O175, respectively, but were not genetically related to these *E. coli* O-serotypes. This finding suggested that some common chemical structures that could not be predicted based on genetic structures are responsible for these cross-reactivities.

### Intra- and inter-species sequence comparison of O-antigen processing-related genes (*wzx*/*wzy* and *wzm*/*wzt*)

**Fig. 3.**
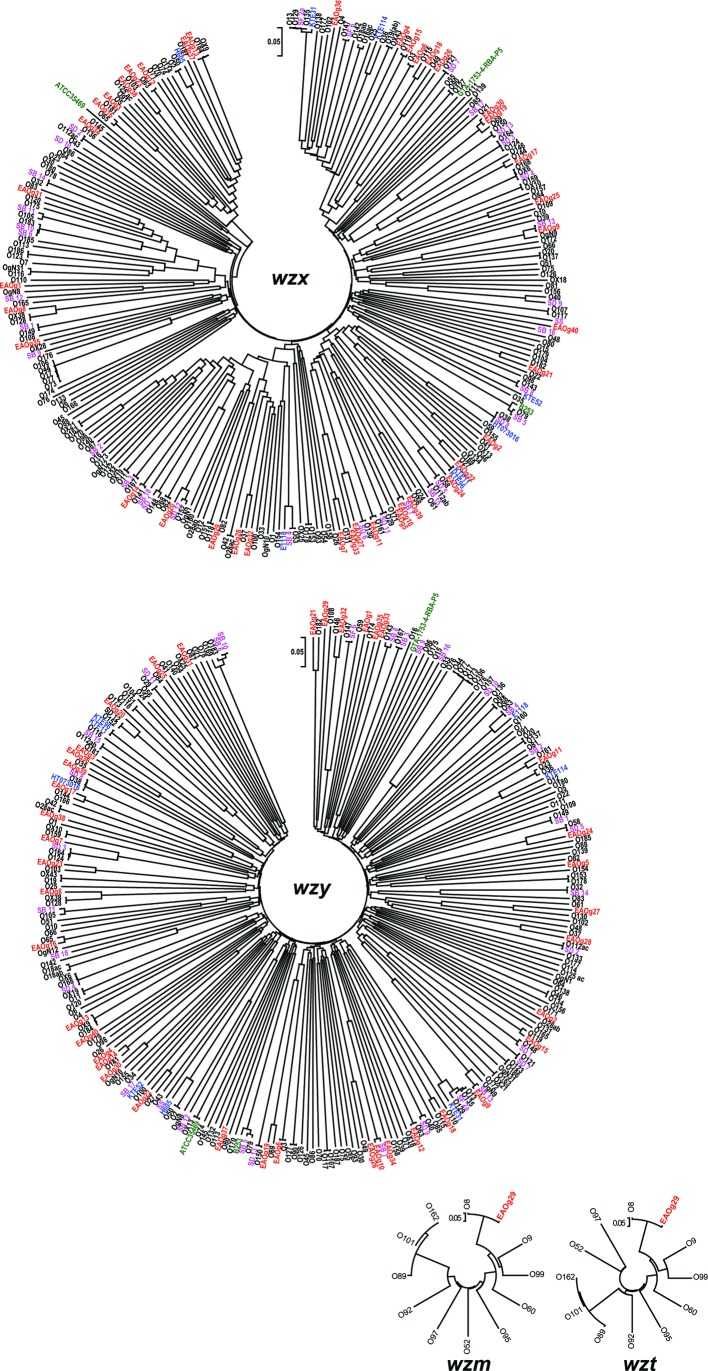
Neighbour-joining trees of the genes for O-antigen subunit translocation and chain synthesis. Neighbour-joining trees were reconstructed based on the sequences of the *wzx*, *wzy*, *wzm* and *wzt* genes from 40 EAOgs (red), 184 known O-genotypes of *E. coli* (black), 34 known serogroups of *Shigella* species (purple), 3 genome-sequenced *E. fergusonii* strains (green) and 8 genome-sequenced strains belonging to cryptic *Escherichia* clades (blue). Bar, the nucleotide substitutions (%) per site.

A pair of O-antigen processing-related genes, *wzx/wzy*, which are essential for O-antigen biosynthesis, were identified in the O-AGCs of all *E. albertii* strains analysed. One strain also contained other types of genes (*wzm/wzt*). As these genes were used for developing a PCR-based O-genotyping system of *E. coli* [41], these *E. albertii* genes may also be usable as targets to develop a similar PCR-based O-genotyping system for *E. albertii *. Therefore, we performed a fine sequence comparison of the *wzx*/*wzy* and *wzm*/*wzt* genes of *E. albertii* with those of known *E. coli */* Shigella* O-serotypes and other *Escherichia* species (Fig. 3, Table S1). This analysis revealed that the *wzx*/*wzy* genes of EAOg6, EAOg9–18 and EAOg30 and the *wzm/wzt* genes of EAOg2 are highly similar (>95 % nucleotide sequence identity) to the genes of *E. coli */* Shigella* counterparts, as expected from the results of whole-O-AGC comparison (Fig. 3, Table S1). Among the remaining 28 EAOgs, although 12 contained the *wzx*/*wzy* genes showing moderate similarity to the genes of *E. coli */* Shigella* or other *Escherichia* species (80–94 % nucleotide sequence identity), the genes of 16 EAOgs shared less than 70 % nucleotide sequence identity with any of the *wzx*/*wzy* genes from *E. coli* and other *Escherichia* species. Interestingly, while the *wzm/wzt* genes of EAOg29 shared more than 98 % nucleotide sequence identity with the genes of *E. coli* O8 as mentioned above, the *wzx/wzy* of EAOg29 showed no significant similarity to the genes of *E. coli* O8, suggesting the exchange of *wzx/wzy* in either the *E. albertii* or *E. coli* O-AGCs.

### Development and evaluation of an O-genotyping PCR system for *E. albertii*

**Fig. 4.**
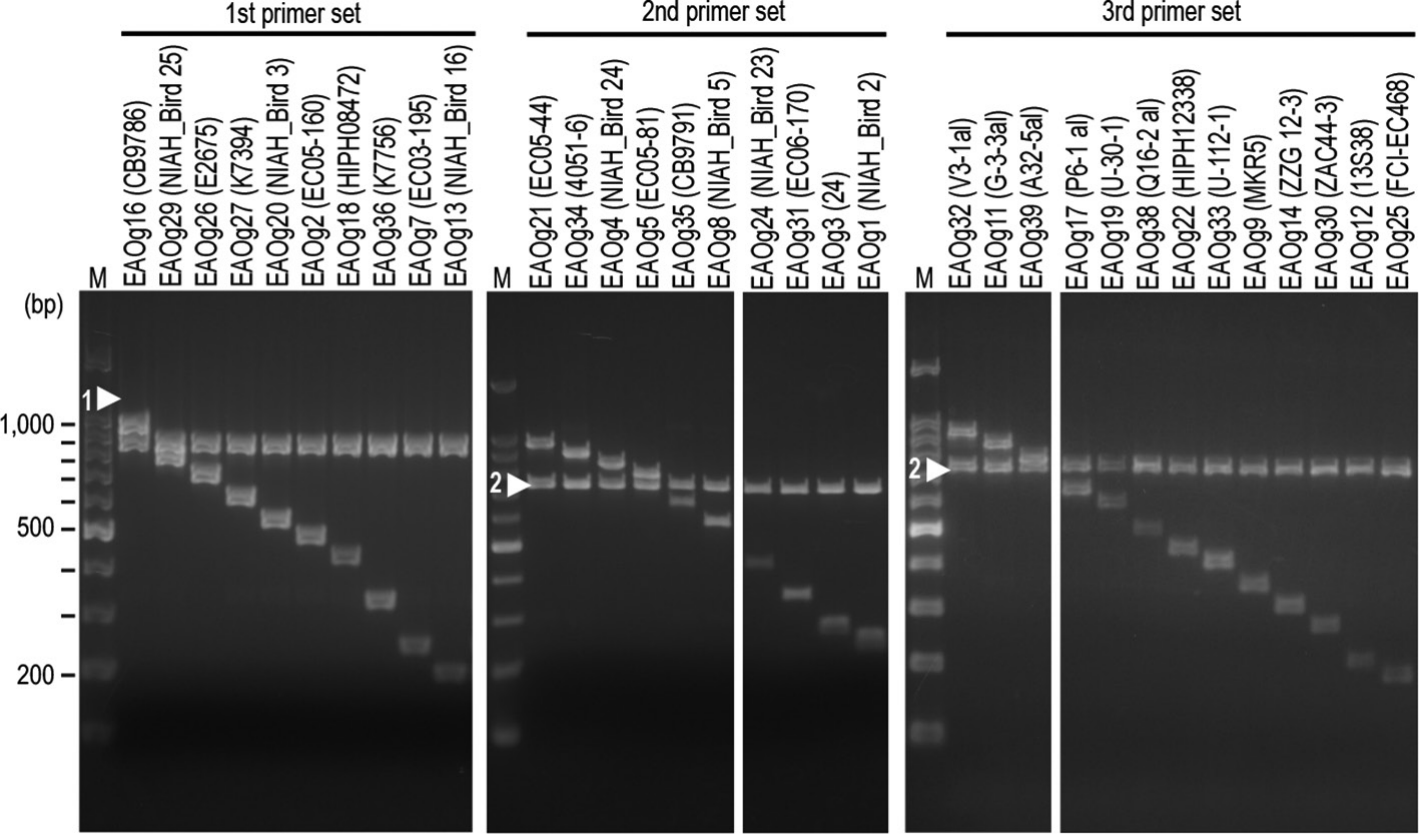
Electrophoresis patterns obtained by EAO-genotyping PCR. A total of 33 strains representing 33 EAOgs were analysed using three sets of PCR primers designed in this study. Strain names are indicated in parentheses. Strains belonging to the remaining seven EAOgs were not available in our laboratory. Arrowheads 1 and 2 indicate the bands derived from the *E. albertii *-specific primer pairs E_al_1_OF/OR and E_al_1_NF/NR, respectively. Lane M, 100 bp DNA ladder.

Although the sequences of some *wzx/wzy* genes of *E. albertii* were highly similar to those of *E. coli *, the *wzx/wzy* genes of the 40 EAOgs were significantly divergent in sequence, indicating that the *wzx/wzy* genes can be used for developing an O-genotyping PCR system for *E. albertii* (EAO-genotyping PCR). Therefore, we designed three multiplex PCR primer sets (Table S5) based on the sequence variation in the *wzx* genes of the 40 EAOgs (Fig. 3, Table S1). Considering the high similarities of several *wzx* genes to *E. coli* genes, we included one *E. albertii *-specific primer pair (E_al_1_OF/E_al_1_OR in the first set, and E_al_1_NF/E_al_1_NR in the second and third sets) [9] in each primer set as a marker to distinguish *E. albertii* from *E. coli* and other species. Using the three sets of PCR primers, we examined the strains representing 33 EAOgs (Fig. 4). The result indicated that the three primer sets yielded PCR products of the expected sizes for each strain. Strains belonging to the remaining seven EAOgs were not available in our laboratory.

To evaluate the performance of the system developed, we determined the O-genotypes of 92 *E. albertii* strains using this system. These strains were isolated from diarrhoeal patients or birds in various regions in Japan. In this analysis, we were able to genotype 76 strains (29 EAOgs; Table S2). Notably, 17 (26.6 %) of the 64 strains isolated from pigeon faeces belonged to EAOg30, although these samples were obtained in five different geographical regions. We were unable to assign 16 strains (17.4 %) to any of the 40 EAOgs. Using the primer sequences, we further performed *in silico* *E. albertii* O-genotyping of an additional 186 genome-sequenced *E. albertii* strains registered in the EnteroBase website. In this analysis, 151 strains were genotyped (25 EAOgs), while 35 strains (18.8 %) were not. Although the ratio of untypable strains was similar to that of the 92 Japanese strains, EAOg4 (37 strains; 19.9 %) and EAOg9 (25 strains; 13.4 %) were predominant in the EnteroBase strains (Table S3). These results indicate that while the system that we developed is useful for O-genotyping of *E. albertii *, the diversity of O-genotypes among *E. albertii* strains is much larger than that captured in this study.

### Distribution of the 40 EAOgs in the *E. albertii* strains genome-sequenced so far and a current phylogenetic view of *E. albertii*

**Fig. 5.**
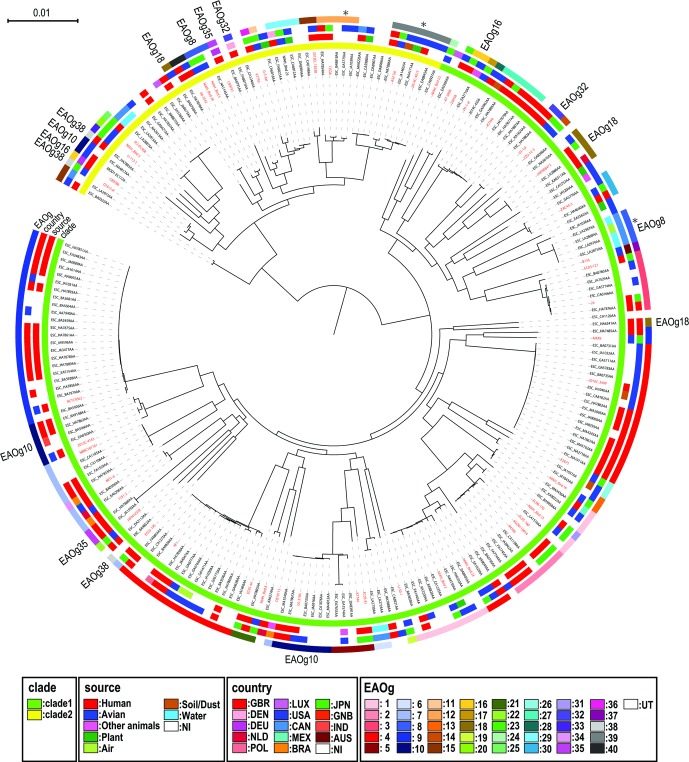
A phylogenetic view of the 225 *E. albertii* strains genome-sequenced so far and the distribution of 40 EAOgs in these strains. An ML tree was reconstructed based on the 94  287 SNPs identified in the core genes (*n*=2128) of 225 *E. albertii* strains genome-sequenced so far. The mid-point rooted tree is shown with strain names at each tip. Strains indicated in red were used for the detailed analysis of O-AGCs. For each strain, clades, sources and geographical locations (countries) of strain isolation, and EAOg are indicated. Seven O-genotypes (EAOg8, EAOg10, EAOg16, EAOg18, EAOg32, EAOg35 and EAOg38) distributed in both clades are also indicated. The EAOgs of three strains marked by asterisks were not determined by *in silico* O-genotyping due to the truncation or lack of the *wzx* sequence in their genome assemblies, but were determined by Illumina read mapping. Bar, the mean number of nucleotide substitutions per site. GBR: United Kingdom, DEN: Denmark, DEU: Germany, NLD: Netherlands, POL: Poland, LUX: Luxembourg, USA: United States of America, CAN: Canada, MEX: Mexico, BRA: Brazil, JPN: Japan, GNB: Guinea-Bissau, IND: India, AUS: Australia, NI: no information, UT: untypable.

Finally, we reconstructed an ML phylogenetic tree based on the core gene sequences to investigate the phylogenetic relationship of the genome-sequenced strains used in this study and the distribution of the 40 EAOgs defined in this study (Fig. 5; note that strains having identical core gene sequences were deduplicated; therefore, 225 strains were included in this analysis). This analysis provided a current phylogenetic view of *E. albertii* and the distribution of the 40 EAOgs in it. Although *E. albertii* strains were divided into two clades (clades 1 and 2) and both clades, particularly clade 2, contained multiple deep branching lineages, clear correlation was not observed between specific O-genotypes and strain sources (isolation sources and geographical locations). Notably, seven O-genotypes (EAOg8, EAOg10, EAOg16, EAOg18, EAOg32, EAOg35 and EAOg38) were distributed in both clades, providing evidence for the intra-species transfer of O-AGCs in *E. albertii* (Fig. 5).

## Discussion

In this study, we analysed the O-AGCs of 65 *E. albertii* strains and defined 40 O-genotypes, named EAOg1–EAOg40. Detailed analyses of the genetic structures and sequences of these genotypes revealed that as many as 25 EAOgs showed notable similarities to the O-AGCs of known *E. coli */* Shigella* serotypes. In accordance with this finding, these EAOgs, except for five EAOgs that could not be examined due to unavailability of strains, showed serological cross-reactivity with corresponding *E. coli* and *Shigella* serotypes. Presumably, the five untested EAOgs also cross-react with the *E. coli */* Shigella* serotypes, to which they showed high similarity. Among the 25 EAOgs, 8 (EAOg6 and EAOg9–15) are particularly interesting because their entire O-AGCs were highly similar, even at the level of nucleotide sequence (most showing >98 % nucleotide sequence identity), to their *E. coli */* Shigella* counterparts. The observed high similarity suggests the recent inter-species transfer of these O-AGCs. A similar finding was also obtained between *E. coli* and *E. fergusonii* [42].

Another notable finding was the cross-reactivity of EAOg8 to *E. coli* O55 and O128. This result is consistent with previous findings that the main chain of O-polysaccharides of EAO8 showed a marked similarity to that of *E. coli* O128, and that the terminal monosaccharide of O-polysaccharide is α-colitose in both EAOg8 and O55 [26]. EAOg29 is also interesting in that it contains both the *wzx*/*wzy* and *wzm*/*wzt* gene sets for O-antigen processing. The latter gene set is very similar to that of *E. coli* O8, and EAOg29 was genetically and serologically similar to *E. coli* O8 (Fig. 3). These findings suggest that the *wzm*/*wzt* gene set is mainly involved in the synthesis of EAOg29 O-antigen. It would be interesting to know whether or how the *wzx*/*wzy* genes are involved in or affect O-antigen synthesis in EAOg29.

We developed an O-genotyping PCR system for *E. albertii* (EAO-genotyping PCR) based on the finding that the *wzx*/*wzy* genes are highly divergent in sequence among the 40 EAOgs. The system is similar to those for *E. coli* [41, 43], but differs in the inclusion of primers to detect *an E. albertii*-specific gene [9] in each primer set. These primers were included because the *wzx* sequences of several EAOgs showed >95 % identity to those of *E. coli/Shigella* serotypes. The PCR products derived from these primers would work as markers to distinguish *E. albertii* from *E. coli* and other *Escherichia* species. The utility of the system was verified by genotyping 92 *E. albertii* strains (PCR-based analysis) and 186 strains (*in silico* analysis) obtained from various sources. However, 51 (18.3 %) of the 278 strains tested could not be assigned to any of the 40 EAOgs. This observation indicates that further analyses, particularly of O-AGCs of strains untyped in this study, are required to understand the diversity of O-AGCs in *E. albertii* and improve the EAO-genotyping PCR method.

Our analysis of the phylogenetic relationship of more than 200 *E. albertii* strains genome-sequenced so far and the distribution of the 40 EAOgs in these strains revealed that there was no clear correlation between the distribution of EAOgs and the geographical locations or sources of strain isolation in the strain set analysed. An important finding of this analysis was that as many as seven EAOgs were distributed in two phylogenetically distinct clades (Fig. 5). This finding suggests that intra-species transfer of O-AGCs may have occurred more frequently in *E. albertii* than in *E. coli* [16, 44, 45].

In conclusion, this study provides what is believed to be the first insight into the diversity of O-AGCs in *E. albertii* and defines 40 EAOgs. We showed the genetic and serological similarity of many EAOgs to O-AGCs of known *E. coli */* Shigella* serotypes, and obtained evidence for the intra- and inter-species transfer of O-AGCs within *E. albertii* and between *E. albertii* and *E. coli *. In addition, based on the nucleotide sequence diversity of the *wzx* genes among the 40 EAOgs, we developed an O-genotyping PCR system for *E. albertii *. Results of the evaluation of this system by PCR or *in silico* genotyping indicate that this O-genotyping PCR system is useful, but further analyses are required to understand the diversity of O-AGCs in *E. albertii *.

## Data bibliography

Ooka T. GenBank, BioProject number PRJDB8401, accession numbers LC494303–LC494359 (2019).

## Supplementary Data

Supplementary File 1
